# Clinical-Cytological Grading in Chronic Rhinosinusitis with Nasal Polyps: An Integrated Framework for Precision Medicine

**DOI:** 10.3390/cells15151426

**Published:** 2026-08-06

**Authors:** Matteo Gelardi

**Affiliations:** Unit of Otolaryngology, Department of Clinical and Experimental Medicine, University of Foggia, Viale Luigi Pinto 1, 71122 Foggia, Italy; matteo.gelardi@unifg.it

**Keywords:** chronic rhinosinusitis with nasal polyps, nasal cytology, Clinical-Cytological Grading, eosinophils, mast cells, asthma, allergy, NSAID-exacerbated respiratory disease, recurrence, biologic therapy

## Abstract

Chronic rhinosinusitis with nasal polyps (CRSwNP) is a heterogeneous inflammatory disease in which type 2 inflammation, epithelial dysfunction, and tissue remodeling determine severity, recurrence, and treatment response. Although molecular biomarkers have clarified disease endotypes, their routine use remains limited by cost, availability, and invasiveness. Nasal cytology offers a simple, repeatable, and minimally invasive method to assess—at the mucosal surface—both epithelial morphology and the dominant inflammatory infiltrate, whether neutrophilic, eosinophilic, mast cell, or mixed. Clinical-Cytological Grading (CCG) integrates the dominant cytological pattern with selected comorbidities, including asthma, allergy, and NSAID-exacerbated respiratory disease (N-ERD), into a weighted clinical-cytological framework. In the founding cohort, the highest relapse association was observed when mixed eosinophil–mast cell inflammation coexisted with asthma and N-ERD. This review discusses the rationale, clinical relevance, and translational applications of CCG in CRSwNP, addressing eosinophilic and mixed mast cell–eosinophilic inflammation, epithelial morphology, disease recurrence, difficult-to-treat phenotypes, biologic monitoring, and the operative dialogue between nasal cytology and histopathology. By linking cytological findings with selected clinical comorbidities, CCG may support biologically informed patient characterization and may prompt targeted mast cell assessment in tissue. However, its prognostic accuracy, incremental clinical value, and role in therapeutic decision-making require independent external validation.

## 1. Introduction

Chronic rhinosinusitis with nasal polyps is a chronic inflammatory disease of the sinonasal mucosa, characterized by nasal obstruction, rhinorrhea, olfactory dysfunction, facial pressure, impaired quality of life, and a marked tendency to recur after medical or surgical treatment [1].

Current international recommendations emphasize that CRSwNP is not a single pathological entity but a heterogeneous syndrome in which clinical phenotypes, inflammatory endotypes, epithelial dysfunction, tissue remodeling, and comorbidities interact to influence disease severity and therapeutic response [1,2].

In recent years, type 2 inflammation has become the principal biological framework for interpreting many severe forms of CRSwNP. Eosinophilic inflammation, local IgE production, IL-4/IL-13 signaling, IL-5-mediated eosinophil survival, epithelial cytokines, and comorbidities such as asthma and NSAID-exacerbated respiratory disease (N-ERD) are recognized as important features of severe and recurrent disease [2,3,4].

This biological understanding has supported the development and clinical use of monoclonal antibodies targeting major type 2 inflammatory pathways, including dupilumab, mepolizumab, and omalizumab [5,6,7].

Despite these advances, the translation of molecular endotyping into routine clinical practice remains incomplete. Molecular biomarkers may provide biologically relevant information, but their use can be limited by cost, technical complexity, availability, and limited repeatability in ordinary rhinology settings. In addition, CRSwNP is a dynamic disease. Its inflammatory profile may change over time, after endoscopic surgery, during topical or systemic corticosteroid treatment, and under biologic therapy.

Biologically guided management of CRSwNP therefore requires tools that are not only informative but also practical, repeatable, and potentially relevant to clinical outcomes.

Nasal cytology occupies a particular position in this scenario. It allows a direct microscopic assessment of the inflammatory cell populations present at the mucosal surface and makes it possible to identify neutrophilic, eosinophilic, mast cell, and mixed patterns [8,9].

The central premise of Clinical-Cytological Grading is that cytological findings should not be interpreted in isolation. In CRSwNP, the possible prognostic meaning of a cellular pattern may be modified by the accompanying clinical context, including asthma, allergy, and N-ERD.

Clinical-Cytological Grading was developed to integrate the dominant cytological inflammatory pattern with selected clinical comorbidities. The first prospective longitudinal study on sinonasal polyposis assessed whether nasal cytology and certain clinical variables (including asthma, atopy, ASA sensitivity, and the association between ASA sensitivity and asthma) could influence post-surgical recurrence, with the aim of developing a Clinical-Cytological Grading system and a prognostic index of relapse [10].

Subsequent studies have explored the clinical applicability of CCG in longitudinal and real-world settings and have provided preliminary support for its potential use in patient stratification [11,12,13]. However, the available evidence remains limited, derives largely from studies conducted within the same research network, and does not yet constitute definitive external validation.

CCG should therefore not be regarded as a simple cytological score or as a conventional clinical severity scale. Rather, it is a pragmatic, clinically derived, and hypothesis-generating framework in which inflammatory cellular patterns and selected comorbid traits are combined to estimate recurrence risk. Its original weights and cut-offs require independent validation and possible recalibration in larger and more diverse populations.

More broadly, CCG reflects a shift from an exclusively anatomical interpretation of CRSwNP toward a biologically informed assessment of the patient. Rather than considering the visible polyp as the disease itself, this approach interprets it as the clinical expression of an underlying interaction among mucosal inflammation, epithelial dysfunction, and comorbid airway disease.

The aim of this review is to critically examine the biological rationale, construction, available clinical evidence, limitations, and potential applications of CCG in CRSwNP. Particular attention is given to its proposed role in recurrence stratification, its relationship with difficult-to-treat disease and biologic therapy, and the complementary interaction between nasal cytology and histopathology.

## 2. Conceptual Basis of Clinical-Cytological Grading

Clinical-Cytological Grading rests on the premise that the severity and recurrence of CRSwNP are unlikely to be explained by a single variable. The framework considers the possible interaction between the local inflammatory cellular pattern and the systemic or airway comorbid context.

The cytological component of CCG identifies the dominant inflammatory cellular profile observed on nasal cytology, including neutrophilic, eosinophilic, mast cell, and mixed patterns. Among the mixed profiles, eosinophil–mast cell inflammation has received particular attention because it may be associated with a more complex inflammatory environment [10,11,12,13,14].

The clinical component of CCG includes selected comorbidities associated with CRSwNP severity and recurrence, namely asthma, allergy, and N-ERD. Within the framework, these variables are interpreted as modifiers of the cytological findings rather than as isolated descriptors. Their contribution to recurrence prediction, however, has not been independently quantified for all components of the original score.

CCG was conceived to extend nasal cytology beyond a purely descriptive assessment by integrating the cellular pattern with selected clinical variables. The microscope identifies the inflammatory cellular profile, while clinical evaluation defines the comorbid context. Their combination generates a recurrence-oriented score. 

The working concept underlying CCG is that recurrence risk may be better characterized when cytological findings are interpreted together with the clinical comorbidity profile.

Patients with a similar endoscopic polyp burden may nevertheless show different cytological and clinical profiles. For example, a patient with a neutrophilic pattern and no major comorbidities differs biologically from a patient with mixed eosinophil–mast cell inflammation associated with asthma, allergy, and N-ERD. 

CCG therefore proposes a structured method for translating these differences into a clinically interpretable score. Its actual predictive accuracy and clinical utility remain to be established in independent populations.

## 3. The Construction of the Score: Grading and Prognostic Index of Relapse

A defining feature of CCG is its explicit additive structure. In the original formulation, clinical and cytological variables were assigned pragmatic weights informed by their observed associations with post-surgical relapse and by their presumed clinical relevance [10]. These weights were not derived from a definitive multivariable prediction model, and most individual components did not reach statistical significance in the founding cohort. The assigned points are summed to generate the CCG score, which was operationally linked to the Prognostic Index of Relapse (PIR) in the original model. This structure has subsequently been applied in longitudinal and real-world settings [11,12], but its weighting scheme has not undergone independent statistical recalibration.

**Figure 1:**
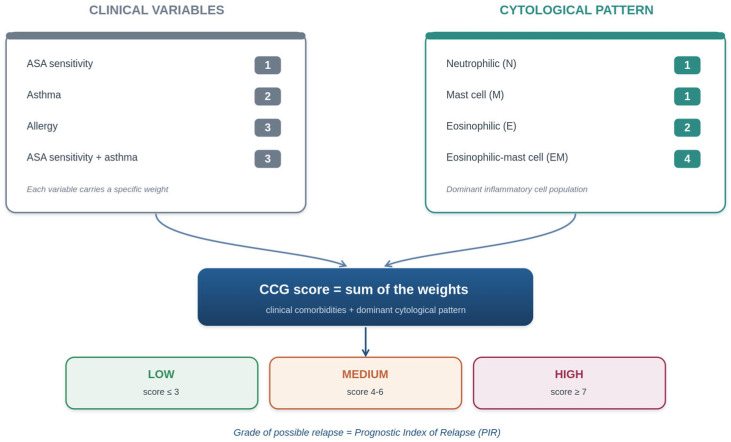
Historical founding scheme of Clinical-Cytological Grading (CCG), adapted from Gelardi et al. [10]. Selected clinical comorbidities and dominant cytological patterns were assigned specific weights in the original model. Their sum generated a low, medium, or high CCG grade and the corresponding proposed Prognostic Index of Relapse. The original 2009 model used the term “ASA sensitivity”, corresponding to the current consensus term NSAID-exacerbated respiratory disease (N-ERD). The figure illustrates the historical construction of the score and should not be interpreted as evidence of definitive validation of the individual weights or cut-offs.

In the original model, the clinical variables were weighted as follows: N-ERD, termed ASA sensitivity in the founding study, 1 point; asthma, 2 points; allergy, 3 points; and the coexistence of N-ERD and asthma, 3 points. The cytological variables were weighted as follows: neutrophilic pattern, 1 point; mast cell pattern, 1 point; eosinophilic pattern, 2 points; and mixed eosinophil–mast cell pattern, 4 points. The sum of the clinical and cytological points generates the CCG score. In the original model, patients were categorized into three CCG grades: low (≤3), medium (4–6), and high (≥7) [10,11]. These concepts should be distinguished. The CCG score is the arithmetic sum of the assigned weights. The CCG grade is the categorical stratification derived from that score. The Prognostic Index of Relapse is the proposed prognostic interpretation attributed to the resulting grade. Although CCG grade and PIR were operationally linked in the founding study, they are conceptually distinct: one is a categorical classification, whereas the other represents its intended prognostic meaning. The original weights and cut-offs are illustrated in Figure 1.

The founding study by Gelardi et al. [10] represented the original CCG framework visually by combining selected clinical comorbidities and cytological patterns within an additive recurrence-oriented scheme (Figure 1).

Two features of this construction deserve emphasis. First, the scoring is cumulative: the coexistence of multiple clinical and cytological traits produces a higher total score. However, a higher score should not be interpreted as a precisely quantified individual probability of relapse, because the original model was not calibrated as a definitive risk-prediction algorithm. Second, the highest single weight was assigned to the mixed eosinophil–mast cell pattern, reflecting its presumed biological relevance and its association with more complex disease configurations in the founding and subsequent studies [10,11,12,13,14].

The statistical basis of the score derives from the founding longitudinal study, in which patients were followed after endoscopic surgery and selected relapse determinants were explored using odds ratios [10]. Only the coexistence of mixed eosinophil–mast cell inflammation, asthma, and N-ERD reached conventional statistical significance in that cohort (OR 4.5; *p* = 0.04). The remaining individual and combined variables showed more modest associations and did not reach statistical significance. Accordingly, the founding study provides statistical support primarily for the highest-risk configuration, whereas most individual score components and their assigned weights remain insufficiently validated. The original CCG should therefore be interpreted as a pragmatic and hypothesis-generating framework rather than as a definitively calibrated prognostic index. These prognostic reference values are summarized in Table 1.

Confidence intervals for the reported odds ratios were not consistently provided in the founding publication and cannot be reconstructed reliably from the published aggregate data alone. For this reason, the present review reports the available odds ratios and p-values without generating post hoc confidence intervals. Future validation studies should report effect estimates with confidence intervals, assess model discrimination and calibration, and determine whether data-driven reweighting of the clinical and cytological components improves the performance of the original score.

Taken together, Figure 1 and Table 1 show that CCG is an explicit additive construct whose highest-risk configuration was supported by a statistically significant association with relapse in the founding cohort. Most other components and combinations were not statistically validated. Their weights therefore remain part of the original pragmatic model and require independent external validation and potential recalibration in larger populations. The conceptual value of the framework lies in interpreting a cytological finding within the accompanying clinical context, whereas the quantitative accuracy of the current weighting scheme remains to be established.

## 4. Clinical Comorbidities as Modifiers of Severity in CRSwNP

CRSwNP is frequently associated with inflammatory comorbidities that may influence disease expression, therapeutic complexity, and recurrence risk. Asthma, allergy, and N-ERD are the principal clinical variables incorporated into the original CCG model. 

The rationale for including these comorbidities is based on the observation that they are not merely background conditions. On the contrary, they identify patients in whom sinonasal inflammation is part of a broader inflammatory phenotype.

Individual comorbidities may be associated with greater disease severity or therapeutic complexity, although their independent prognostic contribution is variable. Their coexistence may identify a clinically more complex phenotype. In a multicenter Italian cross-sectional cohort, higher CCG grades were associated with asthma, N-ERD, allergy, previous surgery, and mixed cytological patterns, whereas lower grades were more frequently associated with fewer comorbidities, fewer previous operations, and a neutrophilic pattern [11]. Because the study was cross-sectional, these associations should not be interpreted as independent confirmation of recurrence prediction.

### 4.1. Asthma and CRSwNP Severity

Asthma is one of the most clinically relevant comorbidities in CRSwNP. The coexistence of asthma and nasal polyposis supports the concept of united airway disease, in which inflammation of the upper and lower respiratory tract represents an interconnected manifestation of a common inflammatory process.

Studies and reviews frequently associate asthma with greater disease severity, recurrence, type 2 inflammation, and therapeutic burden in CRSwNP [15]. Asthma prevalence varies across CRSwNP cohorts, with prospective and real-world studies reporting values of approximately 30–50% [11,16].

Within the CCG framework, asthma is interpreted as a modifier of the cytological context. An eosinophilic or mixed eosinophil–mast cell pattern occurring in a patient with asthma may represent a more complex inflammatory configuration. However, the independent contribution of asthma to the performance of the complete CCG score has not been established in adjusted prediction models.

In patients with concomitant asthma, nasal polyposis may therefore be interpreted within the broader context of airway inflammation.

This broader airway context may contribute to persistent or recurrent mucosal inflammation after medical or surgical treatment. Nevertheless, the magnitude of this contribution and its interaction with the other components of CCG require formal multivariable assessment.

### 4.2. Allergy and CRSwNP: A Context-Dependent Inflammatory Trait

The relationship between allergy and CRSwNP appears more heterogeneous than that observed for asthma or N-ERD. Allergy and atopy have been evaluated extensively in CRS and CRSwNP populations, including in epidemiological studies assessing the prevalence of asthma, N-ERD, and allergic disease [16].

However, the relationship between atopy and CRSwNP severity or recurrence is not always linear. Some studies have questioned whether atopy, by itself, can independently predict disease severity or recurrence [17].

This heterogeneity indicates that allergy should not be interpreted as an isolated or universally applicable prognostic determinant. Within CCG, its meaning is considered in relation to the accompanying cellular pattern and other comorbidities.

For example, an allergic patient without eosinophilic or mixed mast cell–eosinophilic inflammation differs biologically from a patient in whom allergy coexists with asthma, N-ERD, and a mixed cytological pattern. Whether these profiles confer independently different recurrence risks remains to be established.

CCG therefore interprets allergy within the mucosal cellular and comorbidity context. In this framework, allergy is treated as a context-dependent trait rather than as an independently validated predictor. The weight assigned to allergy in the original score requires confirmation and possible recalibration in external datasets.

### 4.3. N-ERD and the Severe-Recurrent Phenotype

N-ERD identifies one of the most aggressive clinical phenotypes of CRSwNP. N-ERD is classically characterized by the association of asthma, CRSwNP, and respiratory reactions to aspirin or other cyclooxygenase-1–inhibiting NSAIDs [18,19].

Patients with CRSwNP and N-ERD typically show a more severe course, greater recurrence of nasal polyps, frequent need for repeated sinonasal surgery, and greater difficulty in achieving durable disease control [20].

Within CCG, N-ERD is considered an important clinical modifier of the cytological context. Its coexistence with eosinophilic, mast cell, or mixed eosinophil–mast cell inflammation may identify a more complex inflammatory phenotype. However, the independent effect of N-ERD and the quantitative validity of its assigned weight have not been established in external multivariable models.

In the founding cohort, the coexistence of N-ERD, asthma, and mixed eosinophil–mast cell inflammation represented the only configuration that reached statistical significance for post-surgical relapse (Table 1). This finding supports attention to the combined phenotype but does not independently validate the weight assigned to N-ERD or its performance in other populations.

### 4.4. Cumulative Effect of Asthma, Allergy, and N-ERD

Asthma, allergy, and N-ERD should not be interpreted as isolated binary variables. Their meaning changes according to their combination and to the underlying cytological profile.

A patient with isolated eosinophilic inflammation differs clinically and biologically from a patient in whom eosinophilia coexists with asthma, allergy, and N-ERD. Similarly, a mixed eosinophil–mast cell pattern occurring together with multiple comorbidities may represent a more complex disease configuration. The extent to which these combinations improve recurrence prediction remains unquantified.

This cumulative logic is central to the original CCG model. The coexistence of multiple traits produces a higher score and is intended to represent a more complex clinical-inflammatory phenotype. However, the original model does not provide a calibrated individual probability of recurrence for each combination.

The working hypothesis of CCG is that increasing convergence between inflammatory cellular patterns and comorbid traits may identify progressively more complex recurrence-risk profiles [10,11,15,18,19,20]. CCG is therefore best described as a clinical-cytological framework that interprets cellular inflammation within the patient’s comorbidity context. Its quantitative prognostic accuracy remains to be established.

## 5. The Cytological Component of CCG

The cytological component of CCG is based on identifying the dominant inflammatory cellular pattern in the nasal mucosal surface. The principal profiles considered in the original framework are neutrophilic, eosinophilic, mast cell, and mixed patterns.

Beyond the inflammatory infiltrate, nasal cytology also provides a direct read-out of the epithelial compartment. On a May–Grünwald–Giemsa-stained smear, the observer can assess ciliated and mucinous cells, recognize signs of epithelial suffering such as ciliary alterations and cytoplasmic changes, and detect immature or metaplastic elements that reflect a disturbed epithelial surface [9,21]. In CRSwNP, where epithelial barrier dysfunction is increasingly recognized as a driver of type 2 inflammation and tissue remodeling [22], these morphological findings add a further layer of information: the same mucosa that harbors an eosinophilic or mixed eosinophil–mast cell infiltrate frequently displays epithelial changes consistent with an activated, poorly regulated barrier. In this sense, nasal cytology captures not only which inflammatory cells are present but also the state of the epithelium that hosts them.

A neutrophilic pattern may reflect infectious, irritative, or non–type 2 inflammatory activity. Although neutrophils are often considered less specific than eosinophils in the context of type 2 CRSwNP, their presence may indicate a different inflammatory environment and influence the interpretation of disease activity [22,23,24].

The eosinophilic pattern is one of the most relevant findings in CRSwNP. Eosinophils are strongly associated with type 2 inflammation, tissue remodeling, mucus alterations, epithelial damage, and the risk of recurrence [14,22,25,26]. Eosinophilic inflammation has therefore become central to the modern interpretation of severe forms of CRSwNP and to the development of biologic therapies.

Mast cells represent an additional component of the inflammatory environment. Their activation may contribute to mediator release, epithelial interactions, vascular permeability, mucus changes, and persistent mucosal inflammation. May–Grünwald–Giemsa-stained nasal cytology can facilitate recognition of mast cells and mixed eosinophil–mast cell patterns. These findings may be under-recognized when routine histopathological assessment relies primarily on hematoxylin–eosin staining [14,24,27,28].

Mixed cellular patterns, particularly eosinophil–mast cell patterns, have received specific attention within CCG. They may represent a more complex inflammatory configuration, especially when accompanied by asthma, allergy, or N-ERD. However, the high weight assigned to this pattern in the original score was not derived from an independently calibrated multivariable model, and its quantitative prognostic contribution requires external validation [10,11].

Studies from the nasal cytology literature have reported mast cell involvement and mixed eosinophil–mast cell inflammation in clinically severe CRSwNP [14,27]. Because several of these investigations originated from related research groups, independent replication remains necessary.

The cytological component of CCG is therefore intended to provide a structured classification of the dominant surface inflammatory pattern. When combined with selected comorbidities, this classification contributes to the original recurrence-oriented score. The reproducibility of pattern assignment and its incremental prognostic value remain to be established formally.

## 6. Beyond an Exclusively Eosinophil-Centered Interpretation: The Contribution of Mast Cells

Histopathology is fundamental to the tissue-level assessment of nasal polyps. Routine hematoxylin–eosin (H&E) staining provides information on tissue architecture, edema, epithelial alterations, fibrosis, basement membrane thickening, glandular changes, and the inflammatory infiltrate. Because eosinophils are readily recognized and have a well-established relationship with type 2 inflammation, they have acquired a central role in the histopathological interpretation of CRSwNP.

To our knowledge, no histopathological grading system has yet been validated that combines inflammatory cell patterns with clinical comorbidities to predict recurrence in CRSwNP. The original CCG model approached this question through nasal cytology by integrating the dominant surface inflammatory pattern with selected clinical variables. This historical observation does not imply superiority of one method over the other, because cytology and histopathology assess different but complementary biological compartments.

The eosinophil-centric model has certainly contributed to the understanding of type 2 inflammation in CRSwNP. However, it also carries an important methodological limitation: it may underestimate clinically relevant cellular components that are not easily recognizable in routine H&E-stained sections. Among these, mast cells are particularly important.

Mast cells are not optimally identified by routine H&E staining. Their reliable recognition usually requires specific histochemical or immunohistochemical methods, such as toluidine blue, Giemsa, tryptase, chymase, and CD117/c-Kit [14,24,28].

Studies comparing cytological and histological assessment in CRSwNP have emphasized that nasal cytology can reveal mast cell inflammation and mixed eosinophil–mast cell patterns, whereas mast cells may remain poorly represented in standard histological reports because of limitations related to staining and magnification [14,27]. Glandular mast cells with a distinct phenotype have also been shown to be highly elevated in nasal polyps when specifically sought [28], and a reappraisal of the role of mast cells in CRSwNP has been advocated [29,30].

These observations have methodological implications. The inflammatory features most readily detected by a routine staining method are also those most likely to be incorporated into clinical classifications. Consequently, an exclusively H&E-based assessment may underrepresent cell populations that require targeted identification. This should be regarded as a limitation of an isolated staining strategy rather than as a limitation of histopathology itself.

This does not imply that histology is less informative than cytology; rather, the two techniques answer different biological questions. Histology provides information on tissue architecture, remodeling, fibrosis, and epithelial alterations, whereas nasal cytology identifies the active inflammatory cell populations present at the mucosal surface. In CRSwNP, these perspectives should be regarded as complementary rather than competitive. Importantly, nasal cytology is particularly effective in detecting mast cells and mixed eosinophil–mast cell inflammation, cellular patterns that may otherwise remain under-recognized in routine histopathological assessment.

By integrating neutrophilic, eosinophilic, mast cell, and mixed inflammatory patterns with key clinical comorbidities, CCG helps move beyond an exclusively eosinophil-centered interpretation of CRSwNP and provides a broader framework for interpreting proposed recurrence risk.

## 7. From H&E to Targeted Mast Cell Assessment: A Proposed Cytologist–Pathologist Handoff

Routine histopathology and nasal cytology should be regarded as complementary sources of information. Histopathology evaluates tissue architecture, stromal edema, epithelial remodeling, basement membrane changes, glandular alterations, fibrosis, and the spatial distribution of inflammatory cells. Nasal cytology provides a surface-level assessment of epithelial morphology and inflammatory cell populations, including neutrophilic, eosinophilic, mast cell, and mixed patterns.

Their combined clinical value may increase when the findings are interpreted together. Nasal cytology may prompt targeted tissue assessment when it shows a predominant mast cell pattern, mixed eosinophil–mast cell inflammation, prominent mast cell degranulation, or persistent mast cell involvement on repeated examinations. In these circumstances, histopathological assessment may be extended beyond routine H&E staining through mast cell-oriented histochemical or immunohistochemical methods, including tryptase, chymase, CD117/c-Kit, toluidine blue, and Giemsa [14,24,28].

This cooperative sequence may be described as a cytologist–pathologist handoff. The clinician provides the relevant clinical and comorbidity profile. The cytologist identifies the dominant mucosal cellular pattern and signals possible mast cell involvement. The pathologist then determines whether targeted staining is appropriate and selects the method according to the diagnostic question.

Tryptase may be used as a principal immunohistochemical marker for confirming mast cell involvement. CD117/c-Kit is sensitive for mast cell identification but is not entirely specific and should be interpreted within the morphological context. Chymase may provide additional phenotypic information on mast cell subsets. Toluidine blue and Giemsa can serve as supportive histochemical methods when appropriate.

Importantly, this is a division of complementary tasks rather than a transfer of competence: the cytologist signals, and the pathologist confirms and characterizes. Both contributions feed a single integrated interpretation.

A proposed practical workflow can be summarized in four steps.

Step 1: Cytological trigger. Targeted mast cell assessment may be considered when nasal cytology shows: (i) a predominant mast cell pattern; (ii) mixed eosinophil–mast cell inflammation; (iii) prominent mast cell degranulation; or (iv) persistent mast cell involvement on repeated examinations.

Step 2: Clinical amplification. The indication may be strengthened when these cytological findings occur in patients with asthma, N-ERD, multiple post-surgical recurrences, previous revision surgery, repeated systemic corticosteroid exposure, or difficult-to-control disease. Allergy may provide additional clinical context but should not be regarded as an independent trigger in the absence of relevant cytological findings.

Step 3: Targeted tissue assessment. Tryptase may be used as the principal immunohistochemical marker for confirming mast cell involvement. CD117/c-Kit may provide sensitive identification but requires morphological interpretation because of its lower specificity. Chymase may support further phenotypic characterization. Toluidine blue or Giemsa may be used as complementary histochemical stains.

Step 4: Integrated interpretation. Mast cell findings should be interpreted together with tissue architecture, eosinophilic infiltration, remodeling, the nasal cytological pattern, and the clinical comorbidity profile. 

**Figure 2:**
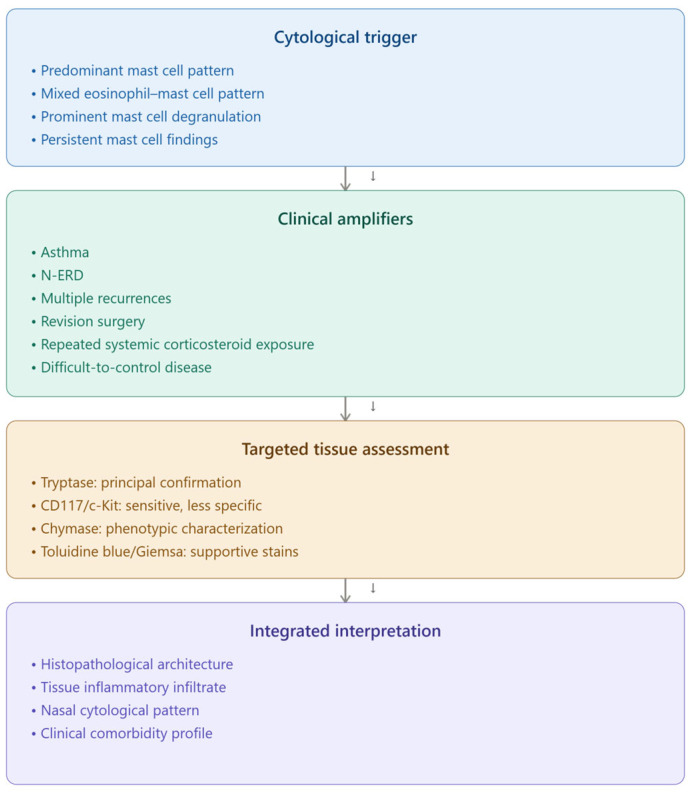
Proposed cytologist–pathologist workflow for targeted mast cell assessment in CRSwNP. A predominant mast cell pattern, mixed eosinophil–mast cell inflammation, prominent degranulation, or persistent mast cell findings on nasal cytology may prompt selective tissue-level assessment. The indication may be strengthened by asthma, N-ERD, multiple recurrences, revision surgery, repeated systemic corticosteroid exposure, or difficult-to-control disease. Tryptase is proposed as the principal confirmatory immunohistochemical marker, while CD117/c-Kit, chymase, toluidine blue, and Giemsa may provide complementary information. The workflow is conceptual and has not yet undergone prospective validation.

Nasal cytology may therefore complement histopathology by identifying surface inflammatory patterns that can prompt selective tissue-level characterization. The proposed workflow is summarized in Figure 2.

## 8. CCG as a Recurrence-Oriented Clinical-Cytological Framework

Recurrence after treatment represents one of the main challenges in the management of CRSwNP. Even after appropriate medical therapy and technically adequate endoscopic surgery, a substantial proportion of patients experience polyp regrowth, symptom relapse, renewed need for systemic corticosteroids, or revision surgery.

This clinical behavior confirms that CRSwNP is not simply a mechanical obstructive disease but a chronic inflammatory condition whose activity may persist even after removal of the polypoid tissue [1,2].

Several risk factors for recurrence have been described in the literature, including asthma, allergic rhinitis, tissue eosinophilia, previous surgery, smoking, and incomplete disease control [25,26,31]. Among these, asthma is repeatedly reported as a significant risk factor for the recurrence of CRS or CRSwNP, while allergic rhinitis and tissue eosinophilia are also frequently associated with postoperative recurrence [23,26,31].

N-ERD identifies an even more aggressive and difficult-to-control phenotype, especially when associated with asthma and nasal polyposis [18,19,20].

The available studies developing, applying, or biologically informing CCG are summarized in Table 2. The table distinguishes studies evaluating the complete CCG framework from those investigating individual cytological or tissue components and from independent studies of nasal cytology during biologic therapy. Evidence supporting an individual component or nasal cytology alone should not be interpreted as validation of the complete CCG score.

The founding longitudinal study followed patients after endoscopic surgery and quantified relapse determinants by odds ratio, defining a clinical-cytological grading system and a prognostic index of relapse [10]. This concept remains clinically relevant, although the original score should be regarded as a pragmatic and hypothesis-generating framework rather than as a definitively validated prognostic model.

CCG differs from models based on single risk factors because it does not consider clinical variables and cytological findings separately. On the contrary, it integrates them. The cytological pattern defines the inflammatory cellular substrate, while asthma, allergy, and N-ERD define the clinical context in which that substrate is expressed. In this way, CCG translates the interaction between local mucosal inflammation and airway comorbid traits into a recurrence-oriented clinical-cytological framework.

This approach is clinically important because the same cytological pattern may not carry an identical meaning in all patients. Isolated eosinophilic inflammation may indicate type 2 mucosal activity, but eosinophilic inflammation associated with asthma and N-ERD sensitivity identifies a more severe airway phenotype. Likewise, a mixed eosinophil–mast cell pattern may acquire particular prognostic relevance when associated with multiple comorbidities. CCG therefore proposes that cytological findings should be interpreted within their clinical context, although the incremental prognostic value of this integration has not yet been established.

The clinical applicability of this model has been explored in subsequent studies. In a five-year longitudinal study of treated patients, stratification by CCG grade tracked disease trajectory and the need for surgery, with markedly higher surgical rates in untreated controls across all grades [8]. In a large real-world cross-sectional study, patients could be stratified by CCG and phenotype, and high-grade CCG was associated with mixed cytology, more comorbidities, and more previous operations [11]. More recent work has further emphasized the relationship between CCG and recurrence prediction [13]. However, these studies were conducted largely within the same research network and do not constitute independent external validation.

No study has yet compared the predictive performance of CCG directly with EPOS/EUFOREA criteria, endoscopic or symptom scores, tissue or blood eosinophilia, previous surgery, or adjusted multivariable clinical models. Therefore, the incremental value of CCG beyond established predictors remains unknown.

From the precision-medicine perspective, the main value of CCG is not simply to classify patients as mild or severe. Its value is to explain why a patient is at risk, because of a dominant eosinophilic pattern, a mixed mast cell–eosinophilic infiltrate, asthma, N-ERD, or the convergence of all these variables. This explanatory dimension may support biological interpretation, but it has not yet been shown to improve therapeutic decision-making or patient outcomes.

Therefore, CCG may be regarded as a recurrence-oriented clinical-cytological framework rather than as a validated biomarker. Unlike molecular biomarkers, which often require specialized laboratory platforms, CCG relies on clinical assessment and nasal cytology, both repeatable in the outpatient setting. This makes it potentially suitable for longitudinal assessment, although its value in monitoring treatment response has not yet been validated prospectively.

A central statement emerges from this model:

The prognostic value of CCG lies in the convergence between inflammatory cellular patterns and comorbid traits. The greater the convergence, the greater the expected risk of recurrence. 

This principle also explains why CCG is fully aligned with current precision-medicine strategies. Modern management of CRSwNP requires not only endotyping but practical tools able to translate endotype-related information into therapeutic decisions. CCG may provide a translational bridge connecting the microscope, the clinical phenotype, and the expected disease trajectory. 

## 9. CCG and Difficult-to-Treat CRSwNP

The concept of difficult-to-treat CRSwNP has become increasingly important in the era of biologic therapies. According to current recommendations, disease severity and eligibility for biologics are assessed through multiple clinical dimensions, including polyp burden, impaired quality of life, loss of smell, need for systemic corticosteroids, previous surgery, and comorbid asthma [1,2]. These criteria remain the established basis for clinical assessment and biologic eligibility, but they do not directly characterize the local cellular pattern of inflammation.

CCG may contribute to the characterization of difficult-to-treat CRSwNP by adding a cellular and comorbid layer to the clinical assessment. Patients with eosinophilic or mixed eosinophil–mast cell inflammation associated with asthma and/or N-ERD may represent a subgroup with greater inflammatory persistence and a higher probability of recurrence. These patients may represent a subgroup of particular clinical interest, although CCG should not currently be used independently to determine surveillance intensity, treatment escalation, or biologic eligibility.

In this context, CCG should not be interpreted as an alternative to the EPOS/EUFOREA criteria. Rather, it should be considered complementary. The EPOS/EUFOREA criteria define clinical severity and biologic eligibility at the guideline level; CCG may provide complementary clinical-cytological information on the inflammatory context of that severity. No prospective study has directly compared CCG with EPOS/EUFOREA criteria or demonstrated that CCG adds independent predictive value after adjustment for established clinical variables, including symptom burden, endoscopic severity, previous surgery, systemic corticosteroid exposure, asthma, N-ERD, tissue eosinophilia, and peripheral blood eosinophilia. Its role should therefore be regarded as complementary and exploratory.

This complementarity is particularly relevant in patients whose clinical picture appears ambiguous. For example, two patients may have similar endoscopic scores but different cytological patterns and comorbid profiles. One may show a neutrophilic pattern without asthma or N-ERD; the other may show mixed eosinophil–mast cell inflammation with asthma and N-ERD. The endoscopic appearance may be comparable, while the underlying inflammatory profiles differ; whether these differences independently predict recurrence or therapeutic needs remains to be established.

CCG may help extend the assessment of CRSwNP beyond visible polyp burden. It supports the idea that the polyp is the macroscopic expression of an underlying cellular and clinical inflammatory network.

## 10. CCG and Biologic Therapies

Biologic therapies have transformed the therapeutic landscape of severe CRSwNP. Dupilumab, mepolizumab, and omalizumab have demonstrated significant efficacy in reducing polyp size, improving nasal obstruction and olfaction, reducing the use of systemic corticosteroids, and improving quality of life in selected patients [5,6,7]. These therapies have also reinforced the need for accurate patient stratification, since not all patients with CRSwNP share the same inflammatory profile, the same risk of recurrence, or the same expected response.

CCG may be investigated in several stages of biologic management. First, it may help identify patients with a high-risk inflammatory phenotype. Eosinophilic inflammation, mixed eosinophil–mast cell patterns, asthma, and N-ERD are all clinically relevant elements that may support the interpretation of severe type 2 disease. When these features converge, they may identify a clinically complex inflammatory phenotype, but CCG should not currently be used independently to determine biologic eligibility or treatment selection.

Second, nasal cytology may be used to document the inflammatory trajectory over time during biologic therapy. Nasal cytology can be repeated during follow-up and may provide information on changes in the inflammatory cellular pattern under treatment. The mucosal inflammatory profile may provide complementary descriptive information alongside symptoms, endoscopy, olfactory assessment, and quality-of-life measures [32,33]. It should be noted, however, that available evidence supports nasal cytology as a monitoring tool during biologic therapy; whether the complete CCG score provides additional predictive or monitoring value in this setting remains to be established. The available studies evaluated nasal cytology rather than the complete CCG framework and did not establish validated thresholds for predicting response, treatment continuation, switching, or discontinuation.

Third, CCG may be investigated in future studies as a possible adjunct to strategies for monitoring long-term biological and clinical control. Prospective studies are required before cellular remission or persistence can be incorporated into therapeutic decision-making.

From this perspective, CCG is not proposed as a substitute for molecular biomarkers or guideline criteria. Instead, it may act as a repeatable and clinically interpretable research framework that is complementary to current assessment. Its proposed strength lies in combining local cellular information with comorbid traits already considered during the clinical assessment of severe CRSwNP.

CCG may potentially contribute to the characterization of high-risk inflammatory profiles and mucosal cellular changes during advanced therapies, but its role in treatment selection and follow-up remains investigational.

Future studies should assess whether baseline CCG predicts response to biologics, whether changes in CCG correlate with clinical improvement, and whether the persistence of mast cell or mixed inflammation identifies partially responsive patients or those at risk of recurrence after therapeutic modulation. These studies should include predefined clinical outcomes, multivariable adjustment for established predictors, blinded cytological assessment, reproducibility analysis, and independent external validation.

## 11. Translational Accessibility of Nasal Cytology

Advanced molecular and cellular technologies, including transcriptomics, proteomics, epithelial cytokine profiling, single-cell technologies, molecular endotyping, and high-throughput platforms have certainly broadened the understanding of CRSwNP pathophysiology. These approaches have clarified the central role of type 2 inflammation, epithelial barrier dysfunction, local immune activation, and tissue remodeling in severe nasal polyposis [4,12,22].

However, their translation into everyday clinical practice remains limited. Many of these technologies require specialized laboratories, trained personnel, substantial resource requirements, complex analysis systems, and infrastructures that are not available in most rhinology clinics. As a result, the gap between molecular knowledge and everyday clinical decision-making remains considerable.

Nasal cytology has comparatively limited technological requirements. It relies on an optical microscope, a routinely available staining procedure, and a minimally invasive sampling method. It can be repeated over time and performed in the outpatient setting [9,21].

The limited technological requirements of nasal cytology support its potential translational accessibility. However, accessibility should not be equated with demonstrated prognostic utility or cost-effectiveness. Its clinical value depends on standardized methodology, reproducible interpretation, and evidence that the information obtained improves prediction or patient management.

CCG follows this principle. It does not compete with molecular biomarkers; rather, it translates mucosal inflammation into a practical clinical language. By integrating neutrophilic, eosinophilic, mast cell, and mixed cytological patterns with asthma, allergy, and N-ERD, CCG provides a repeatable clinical-cytological framework whose prognostic and economic value still require formal evaluation. No formal cost-effectiveness analysis has yet compared a CCG-based strategy with conventional clinical assessment, histopathological biomarkers, blood biomarkers, or molecular endotyping in CRSwNP.

Nasal cytology should therefore be considered complementary to molecular biomarkers and histopathology rather than as a substitute for either. Its potential contribution lies in providing repeatable cellular information through a standardized method. 

## 12. The Training Gap: Why Nasal Cytology Remains Underused

Despite its potential clinical value, nasal cytology remains underused in many countries. One of the main reasons appears to be educational. Nasal cytology seems to remain inconsistently represented in undergraduate and postgraduate medical training and is rarely included as a structured component of residency programs in otolaryngology, allergology, pulmonology, or pediatrics. As a result, many clinicians may complete their training without becoming familiar with nasal sampling, staining techniques, microscopic interpretation, and clinical-cytological correlation.

This training gap has important consequences. If clinicians are not trained to perform and interpret nasal cytology, they are unlikely to consider it part of the diagnostic or investigational assessment of CRSwNP. Likewise, when tissue assessment relies exclusively on routine H&E staining, mast cell involvement and mixed inflammatory patterns may be under-recognized unless targeted histochemical or immunohistochemical methods are used.

Where nasal cytology has been taught systematically, a different scenario has emerged. In Italy, structured ECM-accredited educational programs, including basic, advanced, and combined cytology–surgery courses, have provided a sustained model of training in nasal cytology, releasing official Continuing Medical Education (ECM) credits within the national system. This form of accreditation distinguishes such activities from informal training. However, formal evidence that these educational programs improve interobserver agreement or clinical outcomes remains limited.

Dedicated courses and advanced masters of this kind may contribute to the creation of trained clinical communities, the development of a shared terminology, and the production of clinical studies and international publications. The Italian experience suggests that sustained, structured training may support the transition from individual experience to shared methodology and terminology. Nevertheless, standardized training alone does not establish interobserver reproducibility, which requires formal blinded agreement studies.

This experience suggests that systematic teaching can support the development and dissemination of scientific practice. A grading system can be evaluated reliably only if clinicians are trained to apply it according to standardized procedures. Therefore, the dissemination of CCG requires not only scientific validation but also educational infrastructure: courses, masters, atlases, consensus documents, interobserver reproducibility studies, and digital teaching platforms.

## 13. From Standardized Nasal Cytology to Digital Support for Clinical-Cytological Grading

Clinical-Cytological Grading is built upon nasal cytology, a methodology that has undergone formal standardization through an expert-based Delphi consensus [21]. Standardized criteria now address the principal technical and interpretative steps of the procedure, including the sampling site, smear preparation, staining, microscopic assessment, identification of inflammatory cell populations, and reporting of cytological findings. This methodological framework provides the foundation for the standardized application of CCG across different clinical settings; however, formal multicenter interobserver reproducibility has not yet been established.

CCG does not therefore require the standardization of a new laboratory technique. Rather, it represents the prognostic translation of standardized nasal cytology into an integrated clinical framework in which the dominant inflammatory cellular pattern is combined with asthma, allergy, and N-ERD. The reliability of the grading depends on the correct application of the established cytological methodology and on the accurate collection of the clinical variables included in the score. Standardization is necessary but does not, by itself, demonstrate interobserver agreement.

May–Grünwald–Giemsa staining is particularly relevant because it permits the clear identification of neutrophils, eosinophils, mast cells, and mixed inflammatory patterns. Its ability to reveal mast cells is especially important in CRSwNP, since this cellular component may be underestimated by routine hematoxylin–eosin histology. Nasal cytology and histopathology should therefore be regarded as complementary methods: histology evaluates tissue architecture, edema, fibrosis, remodeling, and epithelial changes, whereas nasal cytology provides a direct cellular read-out of active mucosal inflammation.

The standardization of nasal cytology also creates the methodological conditions for its digital evolution. Digital image acquisition, annotated cytological datasets, artificial intelligence-assisted cell recognition, and telecytology platforms may support training and facilitate multicenter studies [35,36,37,38]. Their ability to reduce interobserver variability and improve reproducibility remains preliminary and has not yet been validated for broad clinical application. These tools should be regarded as supportive rather than as replacements for the cytologist or clinician.

From this perspective, the future of CCG is not dependent on the creation of a new standard, but on the broader implementation of standardized nasal cytology, structured training, and digitally supported interpretation. Its future may therefore be hybrid: grounded in the simplicity of the optical microscope, secured by methodological standardization, and expanded through digital technologies and artificial intelligence.

## 14. Proposed Clinical Role of CCG in the Management of CRSwNP

Clinical-Cytological Grading can be applied at several points along the clinical course of CRSwNP.

At diagnosis, CCG may help characterize different clinical-cytological profiles. A neutrophilic pattern without major comorbidities may suggest a different disease trajectory from an eosinophilic or mixed eosinophil–mast cell pattern associated with asthma, allergy, or N-ERD.

Before surgery, CCG may provide additional information for patient counseling. However, high CCG values should not currently be used as independently validated estimates of recurrence risk. Surgery should in any case be discussed as part of the long-term management of a chronic inflammatory disease rather than as a definitive cure.

After surgery, CCG may be investigated as an adjunct to established clinical follow-up. It should not currently determine follow-up intensity or treatment escalation independently of symptoms, endoscopy, previous surgery, comorbidities, and current guideline recommendations. Consistently, a calibrated, CCG-based medical protocol has been shown to limit systemic corticosteroid exposure and reduce the need for surgery over five years compared with topical treatment alone [8]. However, this study did not provide independent validation of CCG-guided management.

In the future, CCG could also contribute to treatment optimization. If validated prospectively, the change in CCG over time could help identify patients with stable inflammatory control, partial response, or high risk of recurrence. 

Finally, where nasal cytology is not yet available, the CCG rationale could stimulate a more comorbidity-aware histopathological assessment. When informed of asthma, allergy, N-ERD, recurrence history, or difficult-to-control disease, the pathologist may consider targeted assessment of eosinophils and mast cells in addition to routine H&E staining. Such an assessment should be considered complementary and should not be regarded as equivalent to CCG or as a validated prognostic substitute.

## 15. CCG as an Open Platform: Future Perspectives

A further way to understand CCG is to see it not as a closed score but as an open platform. In its current form, it integrates four inflammatory cytological patterns (neutrophilic, eosinophilic, mast cell, and mixed) with three clinical comorbidities (asthma, allergy, and N-ERD). This architecture, however, may be extensible. Its logic assigns weights to biologically meaningful and potentially measurable features and combines them within a recurrence-oriented framework.

This extensibility is what distinguishes a platform from a static score. As new markers reach clinical maturity, they can in principle be incorporated as additional modules without dismantling the existing structure. Cytology-accessible or tissue-accessible biomarkers already explored in CRSwNP—such as Galectin-10 as a correlate of eosinophilic activity [39], or altered MUC1 expression as a correlate of epithelial and secretory dysfunction [34]—illustrate how the grading could be enriched beyond the four founding cellular patterns. Similarly, potential candidate features may include biofilm presence, secretory or barrier indices, and quantitative epithelial descriptors, which could, once standardized, be evaluated as additional components. Any such extension, however, would require prospective validation, recalibration of the variable weights, and confirmation that the expanded model improves discrimination and clinical utility over the original score before it could be adopted in practice. Such an expanded model should be regarded as a next-generation derivative of CCG rather than as an automatic extension of the original score.

Automated recognition and quantification of inflammatory cells on digital nasal cytology could potentially support more objective measurement, reduce operator dependence, and facilitate multicenter studies [35,36,37,38]. However, these applications remain preliminary and have not yet been validated for broad clinical use or for recalibration of the complete CCG score.

A potential future strength of CCG lies in its capacity to incorporate additional validated components.

### Toward a Clinical-Histological Grading

A possible extension of this platform view is the prospective development of a Clinical-Histological Grading, conceived as a distinct tissue-level framework inspired by CCG. At present this remains a conceptual proposal rather than a validated instrument. The idea would be to reproduce, in the histopathological setting, the same integrative logic that underlies CCG: combining the relevant clinical comorbidities (asthma, allergy, and N-ERD) with a targeted assessment of the inflammatory infiltrate, in which eosinophils and, in particular, mast cells are assessed through dedicated histochemical and immunohistochemical methods, including tryptase, chymase, CD117/c-Kit, toluidine blue, and Giemsa, in addition to routine hematoxylin–eosin staining [14,24,27,28]. Such a construct would need to be built and validated from the ground up, with definition of its components, assignment of weights, and demonstration that it predicts recurrence independently and adds value beyond existing histopathological description. If independently developed and validated, a Clinical-Histological Grading could provide a distinct tissue-based framework for centers where nasal cytology is not routinely available. It should not be assumed to reproduce or replace the current CCG system. The proposed CHG should not be considered a histological validation of the current CCG, but a distinct future model inspired by the same integrative rationale.

This platform view also reframes the relationship between simplicity and sophistication. CCG can remain usable with nothing more than an optical microscope in a routine clinic, while simultaneously offering a structure into which advanced biomarkers and computational tools can be plugged as they mature. This potential capacity for expansion requires prospective validation and demonstration of incremental clinical utility.

**Figure 3:**
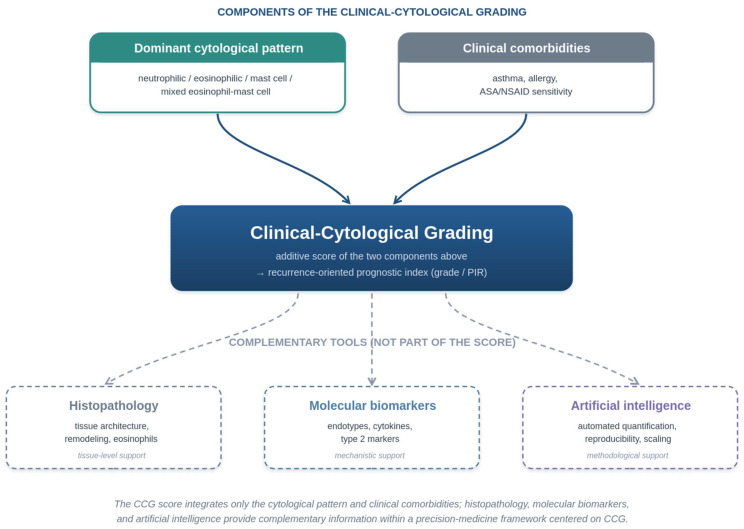
Proposed integrated framework for biologically informed assessment in CRSwNP. The CCG score integrates two components: the dominant cytological pattern and selected clinical comorbidities. Histopathology, molecular biomarkers, and artificial intelligence are shown as complementary sources of information and do not form part of the original score. The figure is conceptual and does not imply that the complete integrated framework has undergone prospective validation.

Figure 3 places this idea in its wider context. Rather than a linear succession in which one discipline replaces another, the evolution of biologically informed management in CRSwNP may be understood as a convergence: CCG integrates the dominant cytological pattern and selected clinical comorbidities, while histopathology, molecular biomarkers, and artificial intelligence may provide complementary layers of information within an integrated framework.

**Figure 4:**
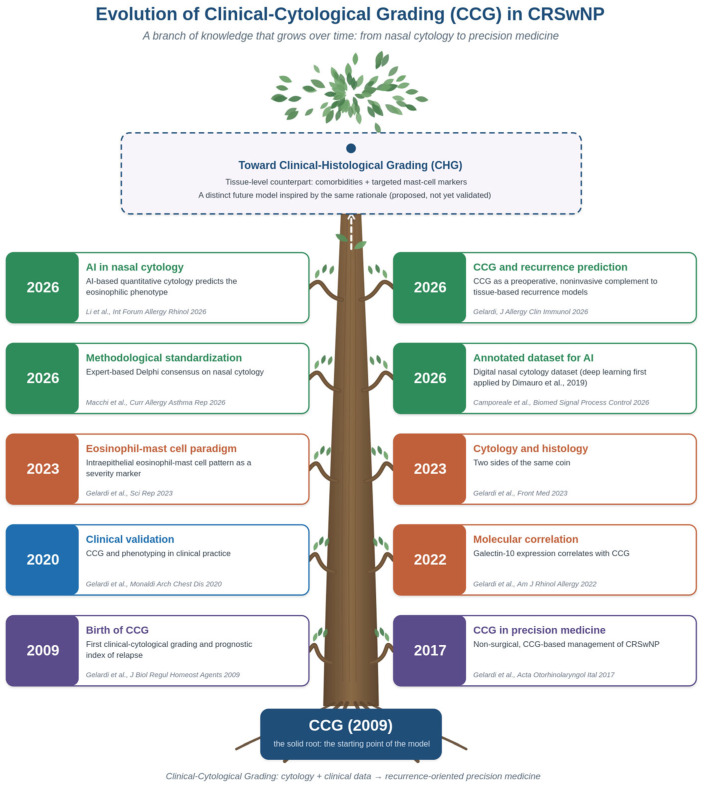
Selected milestones in the development of Clinical-Cytological Grading in CRSwNP. The timeline summarizes the progression from the founding model to subsequent clinical applications, methodological standardization, and preliminary digital and artificial-intelligence-assisted developments. The final node indicates the prospective and unvalidated proposal of a Clinical-Histological Grading. The figure is historical and conceptual and should not be interpreted as evidence of progressive clinical validation of the complete CCG framework.

The same idea can also be read historically. Figure 4 summarizes selected milestones in the development of CCG over time, from its original formulation to subsequent clinical applications, methodological standardization, and preliminary digital developments.

## 16. Current Limitations and Validation Needs

Although the arguments developed in this review support the biological rationale and potential clinical usefulness of CCG, several limitations should be acknowledged. First, the model originated in a single-center, prospective cohort and has been developed and applied largely within the Italian nasal cytology community; its generalizability therefore requires external, international, and multicenter validation in populations with different genetic, environmental, and clinical characteristics [10,11,12,13]. Geographic differences in CRSwNP endotypes, including variable eosinophilic, neutrophilic, type 2, and mixed inflammatory profiles, may affect the performance of the original weights and cut-offs. International validation should therefore include populations with different inflammatory distributions and assess whether geographic recalibration is required.

Second, the reliability of the cytological component depends on operator experience and on correct application of the sampling, staining, and interpretation standards. Even with a standardized methodology, interobserver variability remains a realistic concern, and formal reproducibility studies are needed to quantify it. These studies should use blinded observers and appropriate agreement statistics, including Cohen’s or weighted kappa where applicable. Digital acquisition and artificial-intelligence-assisted cell recognition may help mitigate this dependence, but they do not yet remove it [21,35,36,37,38].

Third, the original weights and cut-offs were derived from a specific cohort in which most individual associations did not reach statistical significance (Table 1). The score should therefore be regarded as a pragmatic and hypothesis-generating framework, whose weights may need recalibration as larger datasets accumulate, rather than as a definitive quantitative algorithm. An additional limitation is the absence of direct comparison with established predictors and clinical frameworks. CCG has not yet been evaluated against EPOS/EUFOREA criteria, symptom or endoscopic scores, previous surgery, tissue eosinophilia, peripheral blood eosinophilia, or multivariable clinical models. Its incremental discrimination, calibration, and clinical utility therefore remain unknown.

Fourth, the evidence linking CCG to the prediction of response to biologic therapy is still limited and largely indirect; prospective studies are required before CCG can be recommended for treatment selection or monitoring in this setting. Existing studies primarily evaluated nasal cytology rather than the complete CCG score. Finally, a conceptual distinction should be kept in mind: demonstrating that CCG is associated with recurrence is not the same as demonstrating that CCG-guided management improves outcomes. Establishing this decision-making utility will require prospective, ideally controlled, studies in which management is actually driven by the grade. These limitations define the research agenda required before CCG can be considered a fully validated prognostic or decision-making tool.

## 17. Conclusions

Clinical-Cytological Grading represents an integrated clinical-cytological framework for interpreting CRSwNP, in which inflammatory cells and clinical comorbidities are considered together. Its strength lies in the combination of two complementary dimensions: the inflammatory cellular pattern observed on nasal cytology and the comorbid clinical profile represented by asthma, allergy, and N-ERD.

In this context, CCG proposes a recurrence-oriented interpretation of nasal cytology by integrating the cellular pattern with selected clinical traits. 

The model also broadens an exclusively eosinophil-centered interpretation of CRSwNP. Although eosinophils remain central to type 2 inflammation, mast cells and mixed eosinophil–mast cell patterns may be underestimated when routine histopathology relies mainly on H&E staining. Nasal cytology, especially when performed with appropriate stains and interpreted by trained clinicians, may facilitate recognition of these cellular patterns and their integration into clinical reasoning. Through the cytologist–pathologist handoff, these findings may also prompt targeted staining with markers such as tryptase, chymase, and CD117/c-Kit. 

In an era of increasingly complex biomarkers, CCG illustrates the potential contribution of accessible and repeatable methods to biologically informed patient assessment. The optical microscope, when combined with standardized methodology, appropriate training, dialogue with histopathology, and digital support, may provide clinically relevant information for patient characterization.

Therefore, CCG may represent a practical framework linking nasal cytology, clinical comorbidities, histopathology, and the proposed assessment of recurrence risk. 

Clinical-Cytological Grading should be viewed as a hypothesis-generating framework that may support future integration among nasal cytology, histopathology, digital pathology, artificial intelligence, and clinical assessment. Independent multicenter validation, reproducibility studies, and prospective evaluation of clinical utility are required before this integrated approach can be recommended for routine patient management.

## Figures and Tables

**Table 1:** Post-surgical relapse associations reported in the founding longitudinal study [10]. Odds ratios and p-values are shown for the available clinical-cytological combinations. Only the coexistence of mixed eosinophil–mast cell inflammation, asthma, and N-ERD reached statistical significance (OR 4.5; *p* = 0.04). The remaining associations were not statistically significant and are reported to document the empirical basis of the original model. The low, medium, and high CCG categories derive from the additive score and should not be interpreted as categories directly estimated from the individual odds ratios. N-ERD corresponds to the condition termed ASA sensitivity in the founding study.

Risk-Factor Combination	Odds Ratio (*p*-Value)	Risk Category
Mixed eosinophil–mast cell + asthma + N-ERD	4.5 (*p* = 0.04)	High
N-ERD + asthma + atopy	1.4 (*p* = 0.23)	Medium
Mixed eosinophil–mast cell	1.6 (*p* = 0.28)	Medium
Mixed eosinophil–mast cell + asthma	1.2 (*p* = 0.81)	Low
Asthma	1.2 (*p* = 0.72)	Low
N-ERD + asthma	1.1 (*p* = 0.82)	Low
Atopy	1.1 (*p* = 0.82)	Low

**Table 2:** Published studies developing, applying, or informing the Clinical-Cytological Grading framework in CRSwNP.

Study	Design, Population, and Setting	Role of CCG or Nasal Cytology	Principal Findings	Main Limitations
Gelardi et al. 2009 [10]	Prospective longitudinal, single-center derivation study; 161 consecutive patients with bilateral nasal polyposis followed after endoscopic sinus surgery.	Development of the original CCG score and Prognostic Index of Relapse.	The combination of mixed eosinophil-mast cell inflammation, asthma, and aspirin sensitivity showed the strongest association with relapse (OR 4.5; *p* = 0.04). Clinical and cytological variables were combined into low-, moderate-, and high-grade categories.	Single-center derivation cohort; most individual associations did not reach statistical significance; pragmatic score weights; no external cohort, calibration, or multivariable validation.
Gelardi et al. 2017 [8]	Longitudinal, non-randomized clinical study; 204 patients, including 145 who regularly followed a CCG-calibrated treatment protocol and 59 controls; 5-year follow-up.	Application of CCG to stratify medical treatment and follow-up.	Patients following the CCG-based protocol showed slower endoscopic progression and fewer surgical procedures. During follow-up, 5/145 treated patients and 29/59 controls underwent surgery.	Non-randomized allocation; groups differed in adherence and treatment exposure; possible selection and adherence bias; same research network; evaluated a management protocol rather than independent prognostic performance.
Gelardi et al. 2020 [11]	Multicenter Italian real-world cross-sectional study; 791 consecutive outpatients from 26 rhinology centers.	Large-scale application of CCG and characterization of clinical-cytological phenotypes.	CCG was low in 26.5%, moderate in 46.3%, and high in 27.2%. High-grade CCG was associated with asthma, aspirin intolerance, allergy, previous operations, and mixed eosinophil-mast cell cytology; mixed cytology occurred in 60.6% of high-grade cases.	Cross-sectional design; no prospective recurrence outcome; no assessment of discrimination, calibration, or incremental prognostic value; conducted within the Italian CCG network.
Gelardi et al. 2023 [14]	Observational cytological-histopathological study; 39 consecutive surgical patients, stratified as low, moderate, or high CCG.	Biological evaluation of mast-cell involvement as a component associated with CCG severity.	Intraepithelial mast-cell counts increased with CCG severity. A threshold of 6 intraepithelial mast cells identified high CCG with an AUC of 0.874, sensitivity of 62.5%, and specificity of 91.3%. Cytology and immunohistochemistry showed agreement in detecting intraepithelial mast cells.	Small surgical cohort; CCG used as the severity reference; mast-cell cytology already contributes to the CCG score, creating possible incorporation or circularity; no prospective recurrence outcome.
Danisman et al. 2023 [32]	Prospective monocentric observational study; 20 patients treated with dupilumab; five assessments over 12 months.	Differential nasal swab cytology during biologic therapy; complete CCG not evaluated.	Nasal eosinophils decreased significantly during dupilumab treatment, together with improvements in polyp score, symptoms, olfaction, and serum IgE. Baseline nasal eosinophilia did not predict clinical response.	Small sample; no control group; swab-based sampling; no complete CCG calculation or CCG-guided treatment; limited predictive analysis.
Piatti et al. 2026 [33]	Prospective monocentric observational study; 45 patients with severe CRSwNP, including 23 treated with dupilumab and 22 with mepolizumab; follow-up to 24 months.	Baseline CCG recorded; longitudinal analysis focused mainly on nasal cytology and clinical response.	Clinical outcomes and nasal eosinophilia improved during both treatments. Eosinophilic and mixed eosinophil-mast cell patterns progressively decreased, while neutrophilic or negative cytology became more frequent. Baseline local eosinophilia correlated with CCG but not with NPS or SNOT-22.	Small single-center cohort; non-randomized biologic selection; concomitant intranasal corticosteroids; CCG not assessed longitudinally as the principal outcome; no validated CCG thresholds for response or treatment modification.
Giancaspro et al. 2025 [34]	Observational tissue-biomarker study; 18 consecutive surgical patients, with 6 patients in each CCG category.	Evaluation of MUC1 expression in relation to CCG-defined disease severity.	MUC1 expression increased significantly from low to high CCG and colocalized predominantly with CD15-positive eosinophils. Eosinophil and tryptase-positive mast-cell counts also increased with CCG severity.	Very small, single-center, selected surgical cohort; CCG used as comparator rather than independently evaluated; no recurrence or treatment-response outcome.

Abbreviations: CCG, Clinical-Cytological Grading; CRSwNP, chronic rhinosinusitis with nasal polyps; OR, odds ratio; AUC, area under the receiver operating characteristic curve; NPS, Nasal Polyp Score; SNOT-22, 22-item Sino-Nasal Outcome Test.

## Data Availability

No new data were created or analyzed in this study. Data sharing is not applicable to this article.
